# Clinically Oriented Classification of Anatomic Variants of the Umbilical Fissure for Ligamentum Teres in the Human Liver

**DOI:** 10.7759/cureus.15460

**Published:** 2021-06-05

**Authors:** Shamir O Cawich, Michael T Gardner, Ramanand Shetty, Patrick Lodenquai, Solange Ramkissoon, Peter Ho, Amanda Chow

**Affiliations:** 1 Surgery, University of the West Indies, St. Augustine, TTO; 2 Anatomy, University of the West Indies, Kingston, JAM; 3 Section of Anatomy, University of the West Indies, Kingston, JAM; 4 Emergency Medicine, University of the West Indies, St. Augustine, TTO

**Keywords:** liver, variant, umbilical, fissure, ligamentum, teres, falicform, pons, hepatis

## Abstract

Background

In the classic descriptions of the human liver, the umbilical fissure (UF) is a long, narrow groove on the visceral surface that receives the ligamentum teres hepatis. In this study, we document the UF variations encountered in a series of cadaveric dissections.

Methods

We reported UF variations using the following classification: Type I refers to "normal" anatomy where there is a long, narrow groove. In type II, the UF was covered by a fibrotic band devoid of hepatic parenchyma. In type III variants, an extension of hepatic parenchyma partially covered but did not obliterate the UF. In type IV variants, the hepatic parenchyma formed a bridge over the UF, completely obliterating the groove. After institutional review board approval, we observed all consecutive cadaveric dissections over five years and recorded the characteristics and dimensions of each UF and its immediate relations.

Results

There were 69 cadavers, and variant UFs were present in 38 (55.1%) cadavers: type II (1.5%), type III (20.3%), and type IV (33.3%).

Conclusions

In this Jamaican population, only 44.9% of persons had conventional "normal" anatomy and 55.1% had UF variants. These variants are clinically significant, as they lead to misinterpretation of patient imaging and can hinder operative procedures on the liver.

## Introduction

In the classic descriptions of the human liver, the umbilical fissure (UF) is a long and narrow groove on the visceral surface that extends from the free margin to the transverse fissure and receives the ligamentum teres hepatis [1-2]. It is also known as the fissure for ligamentum teres [2] or the Rex recess [3].

It is an important anatomic feature for clinicians who treat liver diseases. The UF can be a landmark for radiologists when interpreting imaging studies, and it can also be used to localize the umbilical segment of the left portal vein for vascular assessments. In addition, liver surgeons often use the UF as a landmark during liver resections, and we often grasp the ligamentum teres hepatis to use it as a “handle” to manipulate the liver during laparoscopic liver resections.

Although there have been UF variations described in published literature, we did not encounter a standardized robust classification of these variants. Therefore, we devised a descriptive classification based on our observation of variations in a series of anatomic dissections.

## Materials and methods

Independent researchers observed all consecutive cadaveric dissections during anatomical teaching at the University of the West Indies over a period of five years, from January 1, 2015, to December 30, 2019. Each liver was explanted and closely examined on the dissection bench, with special attention to the characteristics of the UF and its immediate relations: liver segment III, segment IV, transverse fissure, and ligamentum teres hepatis. 

We used classic anatomic description to define “normal anatomy” [1-2]. The UF is classically described as a long, deep groove starting at the anterior margin of the liver and extending posteriorly to the left side of the transverse fissure. The lateral walls are formed by hepatic segments III and IV. It is normally an open groove covered only by a thin layer of Glisson’s capsule, receiving ligamentum teres hepatis at its anterior margin [1-2].

**Figure 1:**
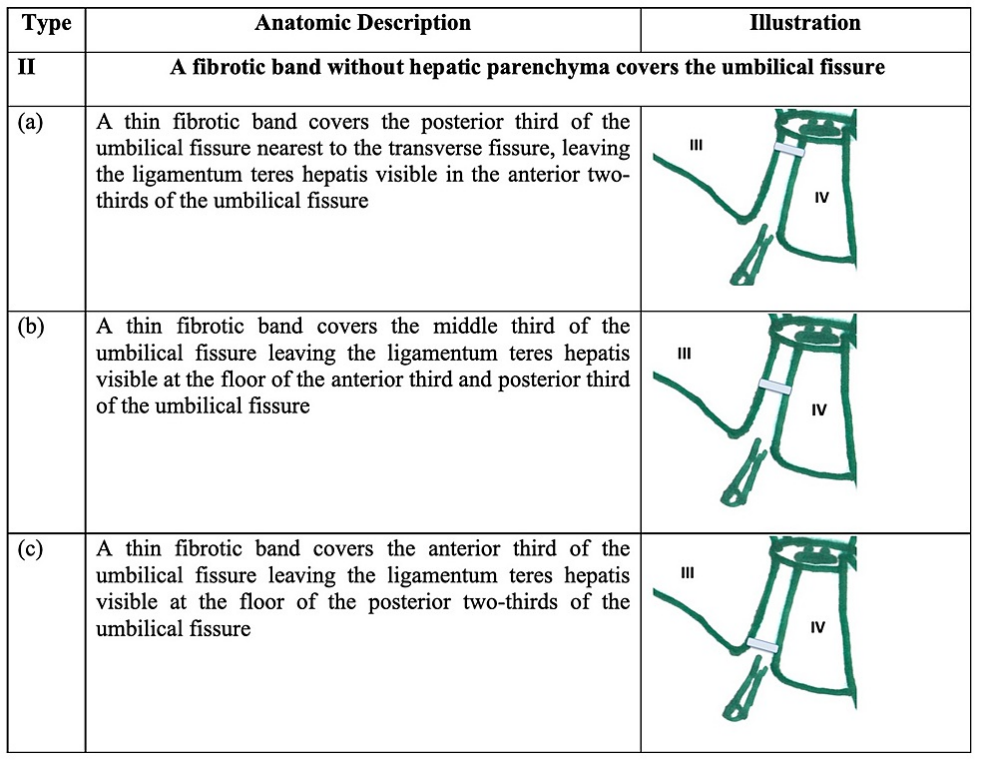
Type II variants A fibrotic band devoid of hepatic parenchyma covers the umbilical fissure.

We devised a descriptive classification based on our observation of variations in a series of anatomic dissections. In this classification, type I refers to “normal anatomy” where there is a long, continuous groove covered by Glisson’s capsule. In type II variants, the UF is covered by a fibrotic band devoid of hepatic parenchyma as illustrated in Figure 1.

**Figure 2:**
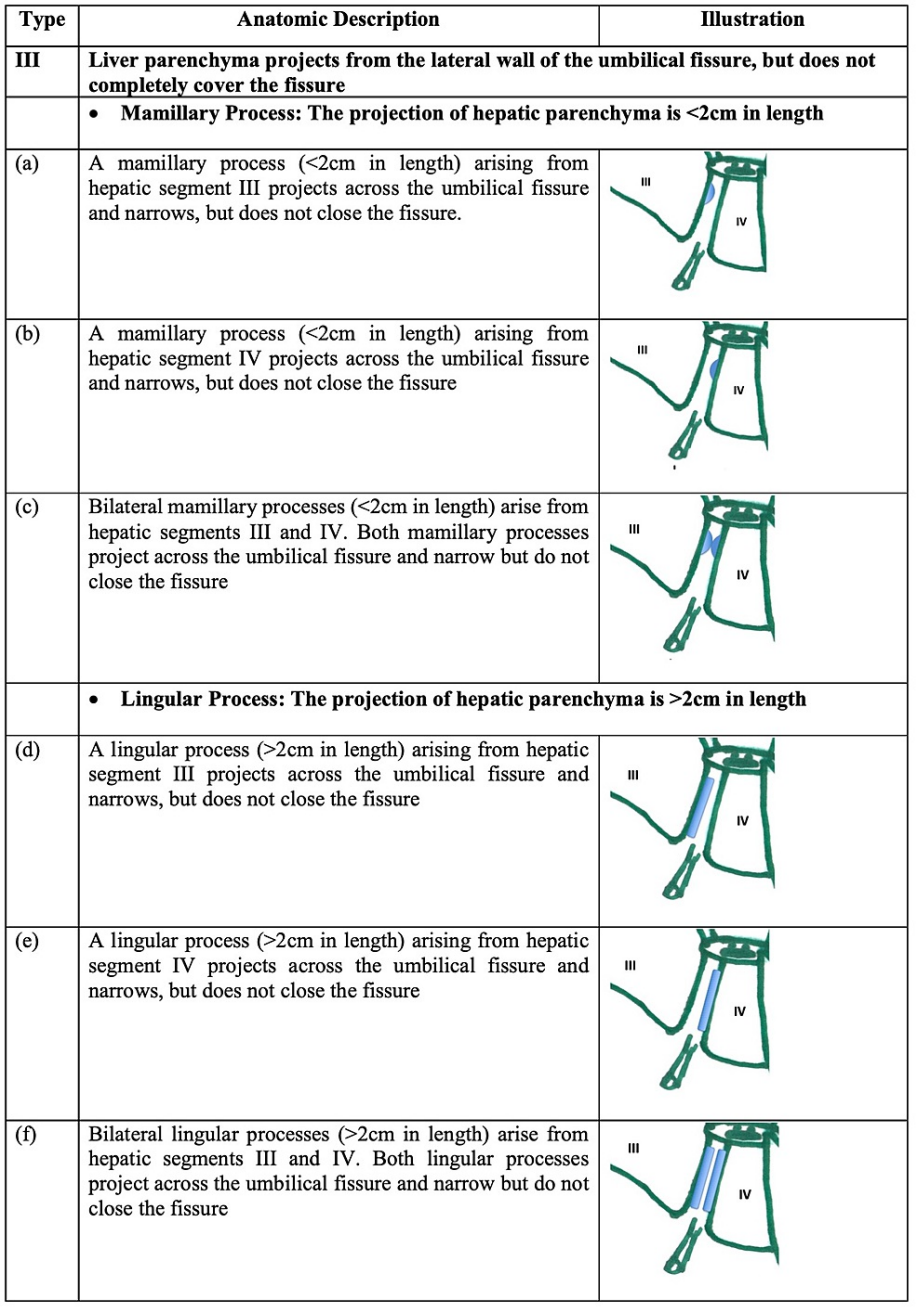
Type III variants Hepatic parenchyma projects from the lateral walls but does not cover the umbilical fissure.

In type III variants, hepatic parenchyma extends over the UF but does not completely cover it as illustrated in Figure 2. We defined two sub-types: projections <2 cm in length were termed “mamillary processes” and those >2 cm in length were termed “lingular processes.”

**Figure 3:**
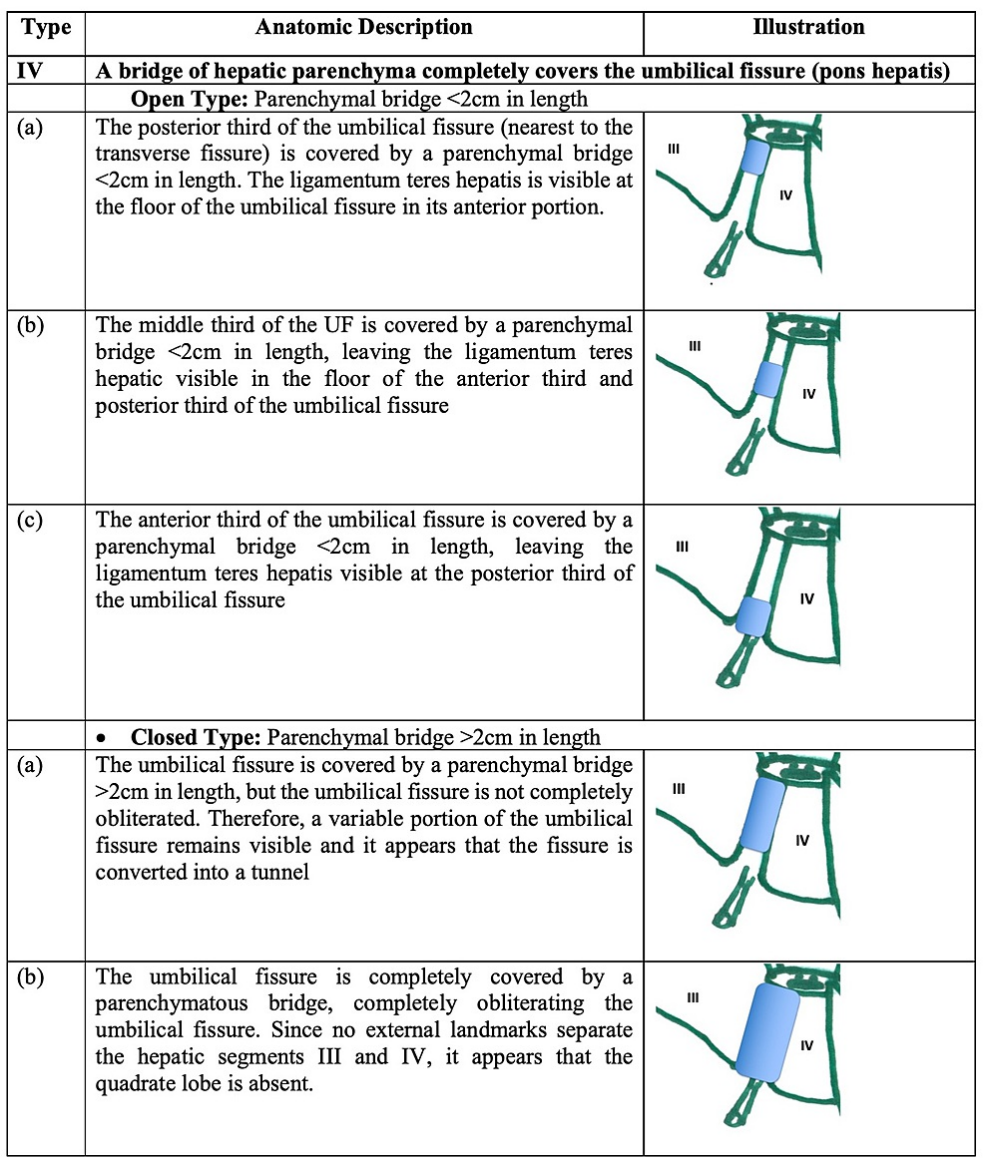
Type IV variants (pons hepatis) A bridge of hepatic parenchyma completely covers the umbilical fissure, converting it into a tunnel.

Figure 3 illustrates type IV variants, where hepatic parenchyma forms a bridge over the UF, completely obliterating the groove. Two sub-types were defined based on the dimensions of the parenchymal bridge: a bridge <2 cm in length was termed an “open-type” UF variant (because it invariably left a portion of the UF visible) and a bridge >2 cm in length was termed a “closed-type” UF variant.

Using these definitions, we described the variations encountered in all consecutive cadavers. Electronic calipers (Mitutoyo ABS Digimatic Caliper Mitutoyo, USA) were used to record the dimensions of the UF and parenchymatous processes and bridges, when present. Two independent investigators recorded the dimensions using electronic calipers and the average was used as the final dimension. The following data were recorded: UF length (distance from transverse fissure to anterior liver margin), UF width (distance from the vertical wall of segments III and IV in a plane parallel to the transverse fissure), UF depth (distance from the visceral liver surface to the deepest point of the UF), parenchymal bridge/process length (distance from transverse fissure to anterior margin of the parenchymatous bridge or process), and parenchymal bridge/process width (distance of the bridge/process extended across the umbilical fissure in a coronal plane). 

## Results

**Figure 4:**
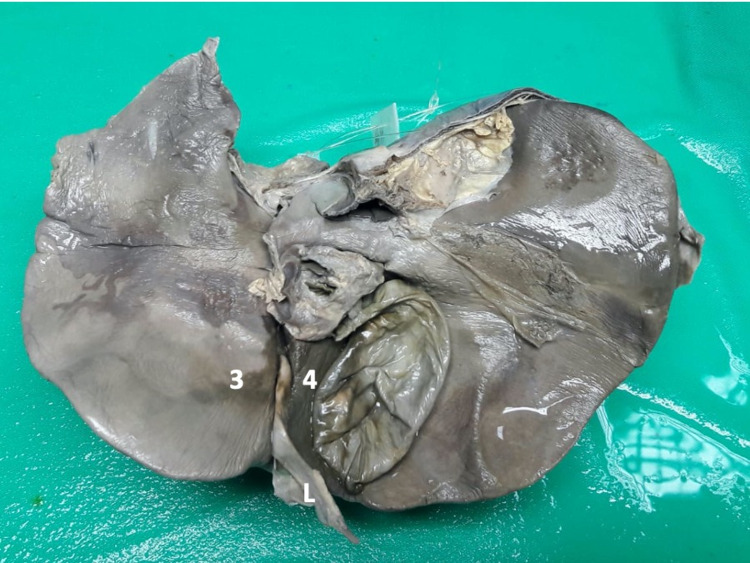
A view of the visceral surface of the liver demonstrating classic anatomic features Type I variant: The umbilical fissure is a continuous groove that commences at the anterior liver edge where it receives the ligamentum teres hepatis (L) and extends to the transverse fissure. It is bounded laterally by hepatic segments III (3) and IV (4).

There were 69 cadavers dissected over the study period, and 31 (44.9%) had a “normal” UF (Figure 4). In those cadavers with “normal” anatomy, the UF had the following dimensions: mean length 51.6 mm (Range 40.9-74.8; Median 49.09; SD +/-9.02), mean width 8.8 mm (Range 4.5-17.3; Median 7.92; SD+/-3.6), and mean depth 17.3 mm (Range 7.7-24.9; Median 17.5; SD +/-5.3). In all cases, the ligamentum teres hepatis entered the UF at the anterior edge of the liver.

There were variant UFs present in 38 (55.1%) cadavers. The prevalence of each variant in our population is outlined in Table 1.

**Table 1:** Prevalence of umbilical fissure variants

Classification of Umbilical Fissure Variants			
Type	Anatomic Description	Illustration	N (%)
I	Continuous, open groove extending from the anterior liver edge to the left side of the transverse fissure		31 (44.9%)
II	A fibrotic band without hepatic parenchyma covers the umbilical fissure		
(a)	A thin fibrotic band covers the posterior third of the umbilical fissure nearest to the transverse fissure, leaving the ligamentum teres hepatis visible in the anterior two-thirds of the umbilical fissure		0
(b)	A thin fibrotic band covers the middle third of the umbilical fissure leaving the ligamentum teres hepatis visible at the floor of the anterior third and posterior third of the umbilical fissure		1 (1.5%)
(c)	A thin fibrotic band covers the anterior third of the umbilical fissure leaving the ligamentum teres hepatis visible at the floor of the posterior two-thirds of the umbilical fissure		0
III	Liver parenchyma projects from the lateral wall of the umbilical fissure, but does not completely cover the fissure		
	Mamillary process: The projection of hepatic parenchyma is <2cm in length		
(a)	A mamillary process (<2cm in length) arising from hepatic segment III projects across the umbilical fissure and narrows, but does not close the fissure.		0
(b)	A mamillary process (<2cm in length) arising from hepatic segment IV projects across the umbilical fissure and narrows but does not close the fissure		1 (1.5%)
(c)	Bilateral mamillary processes (<2cm in length) arise from hepatic segments III and IV. Both mamillary processes project across the umbilical fissure and narrow but do not close the fissure		3 (4.4%)
	Lingular process: The projection of hepatic parenchyma is >2cm in length		
(d)	A lingular process (>2cm in length) arising from hepatic segment III projects across the umbilical fissure and narrows, but does not close the fissure		5 (7.3%)
(e)	A lingular process (>2cm in length) arising from hepatic segment IV projects across the umbilical fissure and narrows, but does not close the fissure		4 (5.8%)
(f)	Bilateral lingular processes (>2cm in length) arise from hepatic segments III and IV. Both lingular processes project across the umbilical fissure and narrow but do not close the fissure		1 (1.5%)
IV	A bridge of hepatic parenchyma completely covers the umbilical fissure (pons hepatis)		
	Open type: parenchymal bridge <2cm in length		
(a)	The posterior third of the umbilical fissure (nearest to the transverse fissure) is covered by a parenchymal bridge <2cm in length. The ligamentum teres hepatis is visible at the floor of the umbilical fissure in its anterior portion.		11 (15.9%)
(b)	The middle third of the UF is covered by a parenchymal bridge <2cm in length, leaving the ligamentum teres hepatic visible in the floor of the anterior third and posterior third of the umbilical fissure		1 (1.5%)
(c)	The anterior third of the umbilical fissure is covered by a parenchymal bridge <2cm in length, leaving the ligamentum teres hepatis visible at the posterior third of the umbilical fissure		1 (1.5%)
	Closed type: parenchymal bridge >2cm in length		
(a)	The umbilical fissure is covered by a parenchymal bridge >2cm in length, but the umbilical fissure is not completely obliterated. Therefore, a variable portion of the umbilical fissure remains visible and it appears that the fissure is converted into a tunnel		9 (13.0%)
(b)	The umbilical fissure is completely covered by a parenchymatous bridge, completely obliterating the umbilical fissure. Since no external landmarks separate the hepatic segments III and IV, it appears that the quadrate lobe is absent.		1 (1.5%)

**Figure 5:**
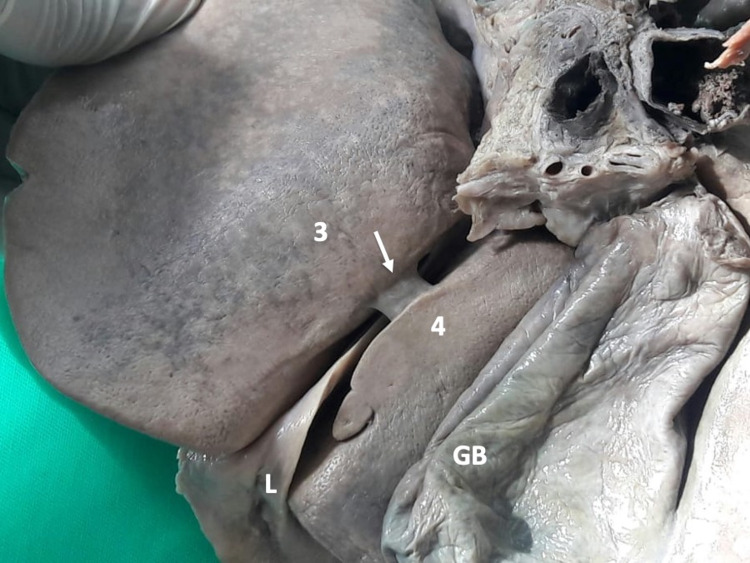
Type II (b) variant In this figure, the ligamentum teres hepatis (L) can be seen entering the anterior end of the umbilical fissure. A fibrotic band (arrow) extends between hepatic segments III (3) and IV (4), covering the middle third of the umbilical fissure.

Type II variants (Figure 5) were the least common in our population. When present, the fibrotic band had a length of 6 mm, a width of 9.1 mm, and a thickness of 3.7 mm.

**Figure 6:**
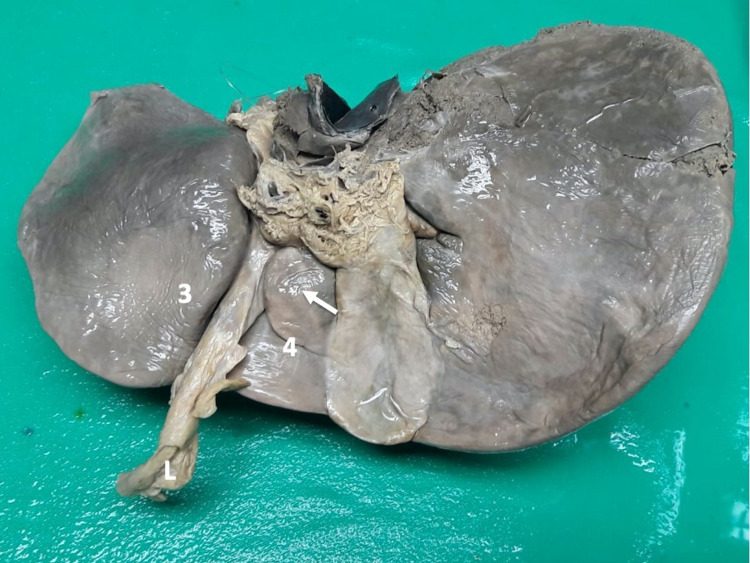
Type III (b) variant In this variant, a mamillary process (arrow) projects from hepatic segment IV (4) to narrow, but not cover, the posterior third of the UF. UF: umbilical fissure; IV: intravenous

**Figure 7:**
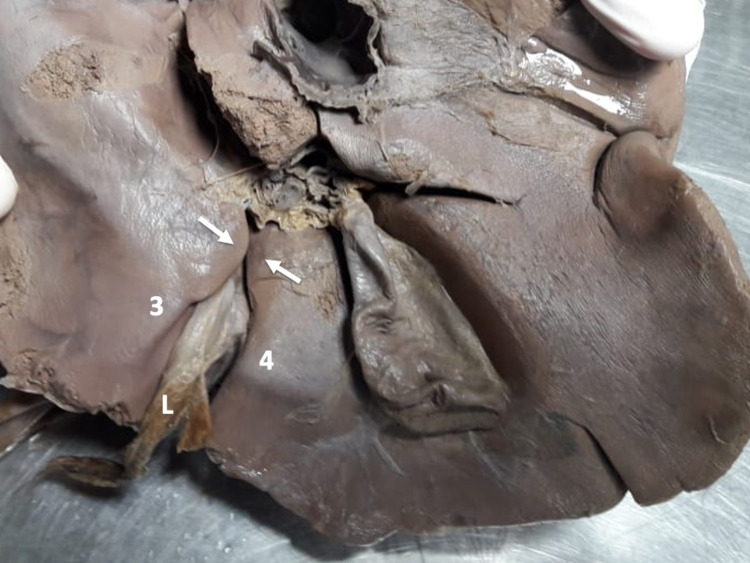
Type III (c) variant In this variant, mamillary processes (arrows) project from hepatic segments III (3) and IV (4) to narrow, but not cover, the UF from both sides. In this variant, the mamillary processes are visible at the posterior third of the UF. UF: umbilical fissure

**Figure 8:**
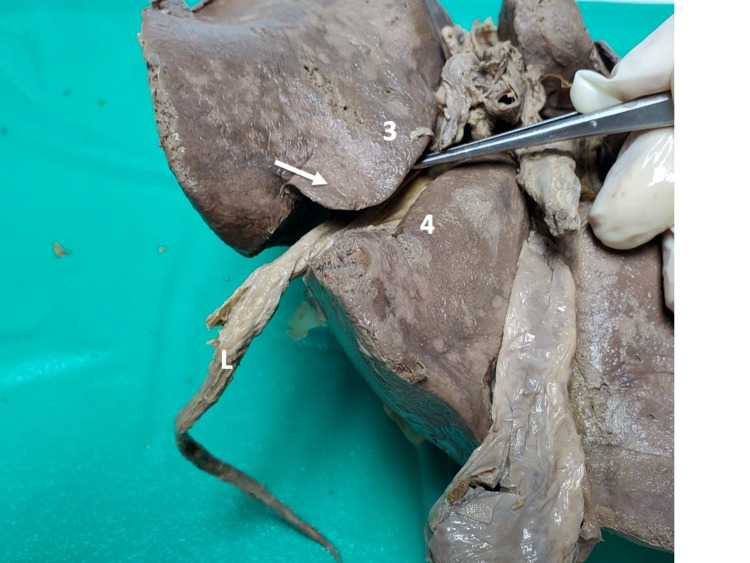
Type III (d) variant In this variant, the ligamentum teres hepatis (L) enters the umbilical fissure. A lingular process (arrow) arises from hepatic segment III (3) and extends across to segment IV (4), narrowing, but not completely covering, the UF. UF: umbilical fissure

**Figure 9:**
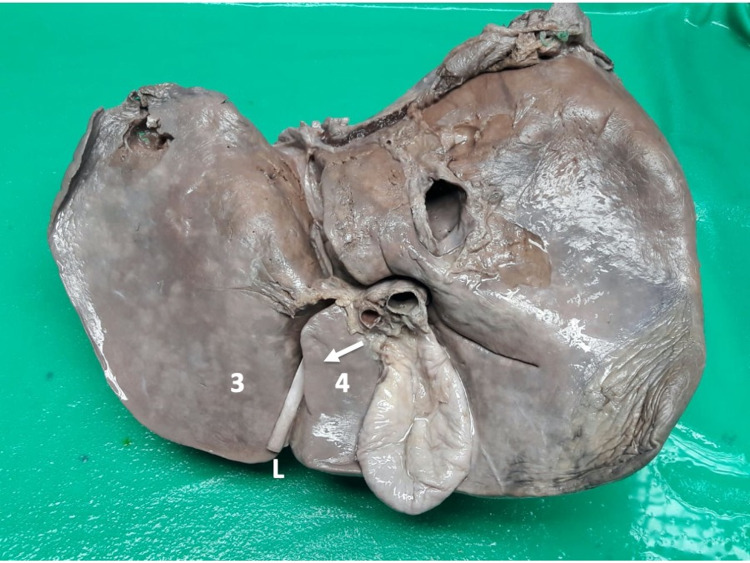
Type III (e) variant In this variant, the ligamentum teres hepatis (L) enters the umbilical fissure. A lingular process (arrow) arises from hepatic segment IV (4) and extends across to segment III (3), narrowing, but not completely covering, the UF. UF: umbilical fissure

**Figure 10:**
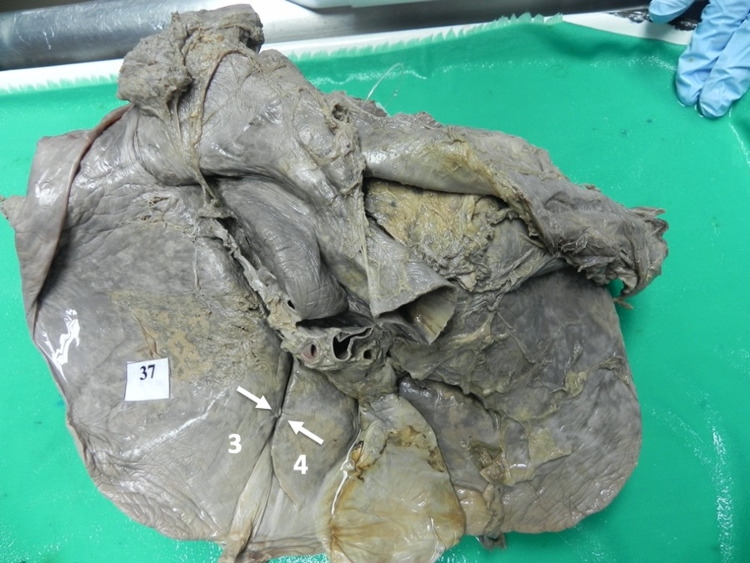
Type III (f) variant In this variant, the ligamentum teres hepatis enters the umbilical fissure. Lingular processes (arrows) arise bilaterally from hepatic segment III (3) and segment IV (4) to narrow, but not completely cover, the UF. UF: umbilical fissure

Type III variants were present in 14 (20.3%) individuals in our population. There were fewer individuals with mamillary processes (Figures 6-7) than lingular processes (Figures 8-10).

**Figure 11:**
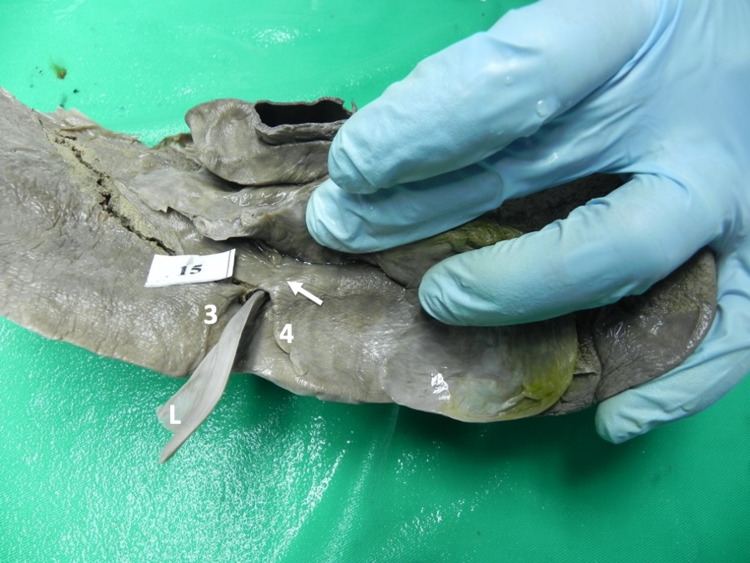
Type IV (a) open variant In this variant, a parenchymal bridge (<2 cm) completely covers the posterior third of the UF (arrow), leaving the anterior UF visible as a deep, long groove that accepts the ligamentum teres hepatis (L). UF: umbilical fissure

**Figure 12:**
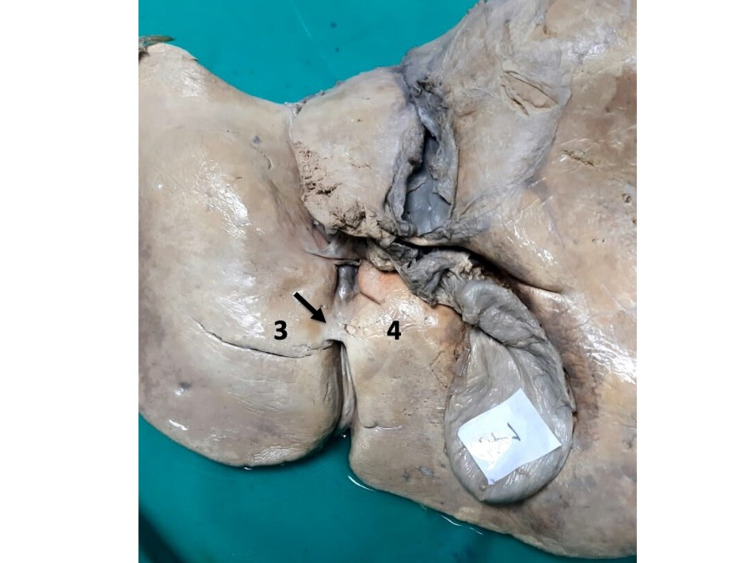
Type IV (b) open variant In this variant, a parenchymal bridge (<2 cm) completely covering the middle third of the UF (arrow), leaving the anterior and posterior thirds of the UF visible as a deep groove. The ligamentum teres hepatis (L) can be seen on the floor of the open segments of the UF. UF: umbilical fissure

**Figure 13:**
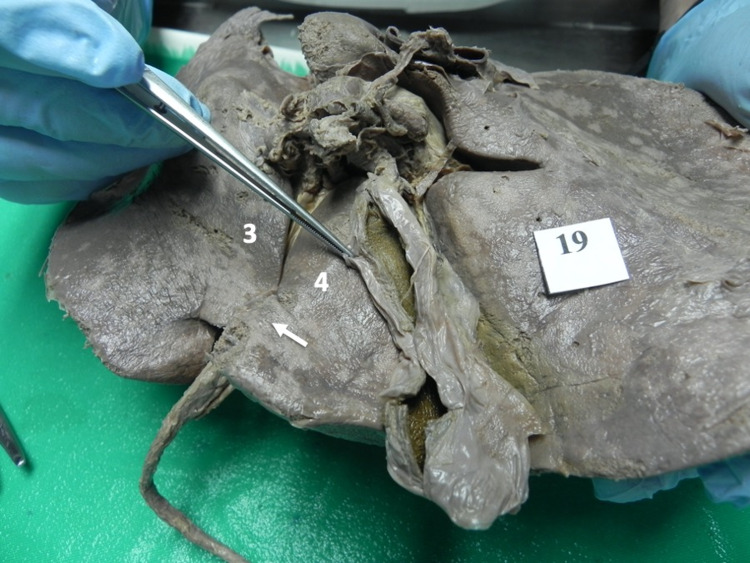
Type IV (c) open variant A parenchymal bridge (<2 cm) completely covers the anterior third of the UF (arrow), leaving the posterior 2/3 of the UF visible as a deep groove. UF: umbilical fissure

Type IV variants were the most common in our population (33.3%). Thirteen (18.8%) individuals had open-type IV variants (Figures 11-13). In these individuals, the parenchymatous bridge had a mean length of 17.02 mm (Range 10.45-19.88; Median 17.41; SD +/-2.53), width of 17.03 mm (Range 6.35-47.75; Median 10.10; SD +/-14.32), and thickness of 9.56 mm (Range 4.63-13.38; Median 9.66; SD +/-3.05).

**Figure 14:**
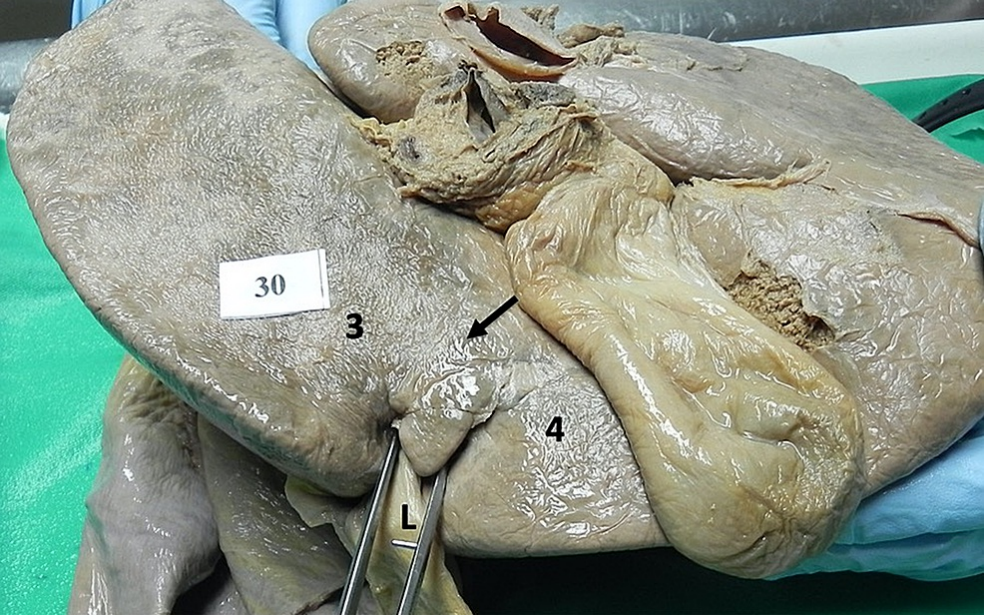
Type IV (a) closed variant A parenchymal bridge (>2 cm) covers the UF (arrow), but the UF remains partially visible (arrow), giving the appearance of the ligamentum teres hepatis (L) entering a tunnel near the anterior liver edge. UF: umbilical fissure

**Figure 15:**
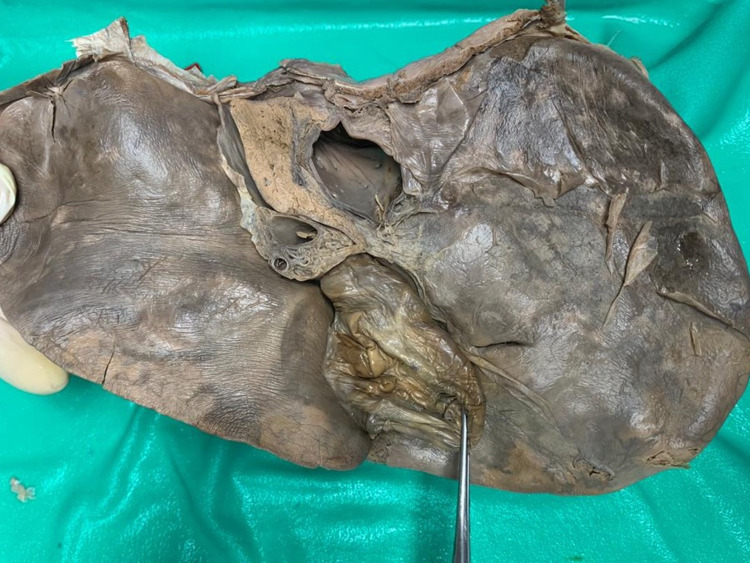
Type IV (b) closed variant A parenchymal bridge completely covers the UF so that no evidence of the UF remains on the visceral hepatic surface. In this image, dissecting forceps retract the gallbladder to the right clearly, demonstrating that there are no surface markings to delineate a quadrate lobe (absent quadrate lobe). UF: umbilical fissure

Ten (14.5%) individuals had closed-type IV variants (Figures 14-15). In these individuals, the parenchymatous bridge had a mean length of 34.66 mm (Range 21.22-66.43; Median 29.45; SD +/-13.10), mean width of 16.98 mm (Range 7.88-47.75; Median 10.47; SD 12.35), and mean thickness of 10.98 mm (Range 6.3-20.15; Median 9.85; Mode 3.60).

## Discussion

The UF is a “normal” groove of the visceral liver surface that extends from the free edge of the liver to the left margin of the transverse fissure [1-3]. In this Jamaican population, 44.9% of individuals had “normal” type I anatomy. 

There have been reports describing variations of the UF [4-22], but a robust classification system was not encountered. We proposed a morphologic classification based on the variations encountered. Interestingly, variant UFs were common in our population (55.1%).

Type II variants were the least common, with a 1.5% prevalence in our population. When present, they were short, thin, avascular membranes that could easily be separated from the underlying ligamentum teres hepatis. They would present little resistance to dissection by liver surgeons performing operations such as a Rex (mesenterico-portal) bypass [8] or cytoreductive surgery with hyperthermic intraperitoneal chemotherapy [13,20].

Type III variants were relatively common, with a 20.3% prevalence in our population. We could find no published data that specifically evaluated these anatomic variants. Mamillary processes were important to recognize as a sub-group because, due to their appearance as localized, irregular projections on cross-sectional imaging, unsuspecting radiologists may confuse them with primary liver tumors [5] or liver metastases [2,23]. By narrowing the UF, type-III lingular variants reduce access to the umbilical segment of the left portal vein, presenting technical difficulty to vascular surgeons performing the Rex shunt and for liver surgeons performing cytoreduction operations with hyperthermic intraperitoneal chemotherapy.

Type IV variants were the most common morphologic variants in our population. These variations have been reported with various names applied to them, such as pons hepatis [5-6,14,16,18,21-22], absent fissure for ligamentum teres [9,12,17] and ligamentum teres tunnel [10-11,15,19]. We propose the use of standardized nomenclature and classifications, possibly those proposed within this paper. In a literature review, we encountered 15 published series (excluding individual case reports) that documented these variations in a combined total of 5,550 individuals [4-6,13-14,16-22,24,25-26]. With the proviso that comparisons are difficult due to varied nomenclature, the documented incidence of type IV variants ranges from a low of 1.25% [17] to a high of 27.8% [6]. The incidence of type IV variants in our population (33.3%) was closer to that documented by Joshi et al. in North India [6].

As liver surgeons, we found the closed-type IV variants interesting. We proposed the use of a parenchymal bridge length of >2 cm to define the closed-type variant because such a large bridge would be clinically significant, in terms of both diagnostics and operative intervention. From a diagnostic point of view, the long and thick hepatic bridge makes Doppler assessment of the umbilical segment of the left portal vein difficult when preparing for Rex shunts between the superior mesenteric vein and the umbilical branch of the left portal vein [8]. In our population, 14.5% of individuals have a closed type variant.

Of particular interest was the variant in which hepatic parenchyma completely obliterated the UF, giving the appearance that the quadrate lobe was absent. In the published literature, similar variations have been described using different names. This variant has been described as a “liver not divided into lobes on the visceral surface” by Kale et al. [7], as “tissue of pons hepatis leaving no boundaries for quadrate lobe” by Anbumani et al. [22], as a “complete absence of a quadrate lobe” by Ebby and Ambike [10] and Joshi et al. [6], and as “absence of a left lobe” by Abdullahi et al. [9], Satessha et al. [12] and Aktan et al. [4]. This variant was present in 1.5% of individuals in our series. Aktan et al. [4] reported that this variant was present in 3.2% of 437 individuals and Joshi et al. [6] documented its presence in 4.4% of 90 individuals. To demonstrate the problem without standardized nomenclature, we refer to the paper by Anbumani et al. [22] in which 3/30 (10%) of individuals were reported to have “no boundaries between left and quadrate lobes,” but published photographs reveal that two of these were actually open-type IV-b and closed-type IV-a variants. Hence, the true incidence of closed-type IVb variants would have only been 3.3% (1/30) instead of 10%, according to our classification system.

Liver surgeons often use the umbilical fissure as a landmark to plan liver resections [2], and this is rendered impossible with a closed type IV variant. In addition, during laparoscopic liver resections, we often grasp the ligamentum teres hepatis with laparoscopic instruments, using it as a “handle” to manipulate the liver. This maneuver now becomes dangerous in the face of this variant because the overlying hepatic parenchyma can be lacerated due to shear stress, leading to significant bleeding from the lacerated parenchyma.

## Conclusions

In this Caribbean population, only 44.9% of persons had conventional "normal" anatomy of the umbilical fissure. There were UF variants in 55.1% of persons in this population with the following distribution: Type 2 (1.5%), Type 3 (20.3%), and Type 4 (33.3%). These variants bear clinical significance, as they can be misinterpreted for liver tumors and hinder sonographic assessment of the umbilical segment of the left portal vein. In addition, the type III and IV variants negatively impact surgical procedures such as hyperthermic intra-peritoneal chemotherapy, Rex shunts, and laparoscopic liver resections.
